# Five Traditional Chinese Medicine External Treatment Methods Combined with Mecobalamin for Diabetic Peripheral Neuropathy: A Network Meta-Analysis

**DOI:** 10.1155/2022/4251022

**Published:** 2022-12-16

**Authors:** Lijuan Xu, Deng Zang, Hui Li, Amanguli Sulitang, Yinhui Li, Jing Ma, Kai Wang, Li Ma

**Affiliations:** ^1^Traditional Chinese Medicine Hospital Affiliated to Xinjiang Medical University, Urumqi, China; ^2^College of Medical Engineering and Technology, Xinjiang Medical University, Urumqi, China

## Abstract

**Background:**

Diabetic peripheral neuropathy (DPN) is one of the most common chronic complications of diabetes. Traditional Chinese medicine (TCM) external treatment has been widely used in China as adjunctive treatment, and some small sample clinical studies have proved its effectiveness. However, due to the limited number of studies, we used network meta-analysis to compare the effectiveness of 5 commonly used external treatment methods of traditional Chinese medicine in the treatment of diabetic peripheral neuropathy.

**Methods:**

We searched PubMed, EMBASE, The Cochrane Library, Web of Science, CNKI, CBM, WanFang Knowledge Service Platform, and VIP databases and collected and screened randomised controlled trials on the external treatment of traditional Chinese medicine combined with mecobalamin in the treatment of DPN according to the inclusion and exclusion criteria. The search period was from 2011 to May 2021. The quality of included studies was assessed using the revised Cochrane risk-of-bias tool for randomized trials. The outcome indicators are Toronto score, median nerve sensory conduction velocity, and median nerve motor conduction velocity.

**Results:**

A total of 22 publications were included in the study. The results of the network meta-analysis showed that acupuncture combined with mecobalamin was superior to other TCM external treatments combined with mecobalamin in terms of decreasing the Toronto score (MD = −2.8, 95% CI: −5.2∼−0.49), improving median nerve sensory conduction velocity (MD = 3.6, 95% CI: 2.4∼4.9), and median nerve motor conduction velocity (MD = 4.5, 95% CI: 2.6∼6.5). The SUCRA value and probability ranking chart showed that among the three outcome indicators, acupuncture combined with mecobalamin was the best, followed by acupoint injection combined with mecobalamin.

**Conclusion:**

In this network meta-analysis, acupuncture combined with mecobalamin shows the best results in the treatment of DPN, followed by acupoint injection combined with mecobalamin.

## 1. Introduction

At present, the prevalence rate of diabetes mellitus (DM) in people aged 18 and above in China is 11.2% [1]. With the prolongation of the course of diabetes, the prevalence of DPN can be as high as over 90% [2] and the incidence of DPN among hospitalized DM patients in China is 60.3% [3]. The pathogenesis of DPN is currently considered related to insulin deficiency or insulin resistance, hyperglycemia, and dyslipidemia [4]. The clinical manifestations of DPN are mainly distal symmetrical neuropathy, which may cause numbness, pain, hyperalgesia, formication, muscle weakness, and other symptoms that seriously affect the quality of life [5]. At present, evidence supports the use of specific anticonvulsants and antidepressants for pain management in patients with diabetic peripheral neuropathy, but effective therapies are not available for many patients, so the treatment of DPN is challenging [6]. External TCM treatments are guided by the holistic view of TCM, and treatments are based on syndrome differentiation. TCM uses drugs or physical therapy to act on human skin, orifices, and acupoints to help qi and blood flow, to relax and dredge the main and collateral channels, and to reach the viscera from outside to inside [7]. With the progress of modern science and technology, the advantages of TCM external methods in the treatment of DPN are being gradually highlighted. Firstly, TCM external treatments are absorbed into the body through the skin and mucous membranes or through the body surface acupoints, to stimulate the conduction of the meridians to help qi to regulate blood flow to the viscera, to avoid the liver effect, and to reduce the burden on the liver and kidneys. Secondly, TCM external treatments are relatively simple to operate, are cost-effective, and can directly reach the lesion with concentrated efficacy [8]. Traditional Chinese medicine external treatment includes acupuncture, massage, fumigation, acupoint application, foot therapy, ear acupoint therapy, and many other methods [4]. At present, more and more RCT studies have confirmed that TCM external treatments play a key role in the treatment of DPN [9–12]. Although much research has been carried out on all kinds of TCM external treatment to evaluate the therapeutic effect of DPN, the results of the research outcome indicators differ and RCT studies are of mixed quality. It is difficult for clinicians to find a high level of evidence and to choose among the numerous TCM external treatments. It is therefore necessary to use statistical methods to compare the TCM external treatments for clinical decision-making. Network meta-analysis is an extension of meta-analysis, which aims to solve the problem of the effectiveness of a certain treatment for a certain disease. Network meta-analysis can solve some problems that may exist in the traditional meta-analysis, such as the lack of available direct comparative evidence and the existence of multiple interventions. Through the method of network meta-analysis, the effectiveness of different treatment methods for the same disease can be systematically compared [13]. It can compare the efficacy of three or more treatments and provide an integrated comparison of the pros and cons of various interventions against one disease [14]. At present, most of the external treatment methods for DPN reported in the literature focus on acupuncture, TCM foot bath, acupoint application, acupoint injection, and TCM fumigation. Hence, in this study, the network meta-analysis method was used to analyze these five TCM external treatment methods in current clinical use and to provide more precise evidence to support TCM external treatment methods of DPN.

## 2. Methods

### 2.1. Data Sources and Search Strategy

Chinese databases, including CNKI, CBM, WanFang Knowledge Service Platform, and VIP, and English databases, including PubMed, Cochrane Library, Embase, and Web of Science, were searched by computer. The searches took place from 2011 to May 2021. The corresponding Chinese subject words were retrieved from the CBM database, the Chinese free words were retrieved from the VIP database, and the corresponding English subject words and free words were retrieved from PubMed. The complete search strategy is shown in Appendix 1 of Supplementary Materials (available here). We followed the preferred reporting items for systematic reviews and meta-analyses guideline (PRISMA-2020) [15] and the extension statement for network meta-analysis (PRISM-NMA) [16].

### 2.2. Inclusion and Exclusion Criteria

Inclusion criteria for the study were as follows: (1) Study type: randomized controlled trial (RCT). (2) Subjects: patients who met the DPN diagnostic criteria of the 2020 guidelines for the prevention and treatment of type 2 diabetes in China, regardless of disease course and source of cases. (3) Intervention measures: the experimental group used one TCM external treatment (TCM fumigation, TCM foot bath, acupoint application, acupoint injection, and acupuncture) combined with mecobalamin treatment. The control group was treated with mecobalamin alone. In the relevant study, the dose of mecobalamin remained the same. The basic treatment (e.g., glucose control drugs, diabetic diet, and exercise guidance) had to be the same in each group in the relevant study and was considered as a basic intervention. (4) Outcome indicators: one of the following outcomes was clearly reported in the literature: ① Toronto score; ② median nerve sensory conduction velocity; ③ median nerve motor conduction velocity. (4) Chinese and English literature published between January 1, 2011, and May 12, 2021.

Exclusion criteria for the study were as follows: (1) the research type is not clearly stated, (2) the effective outcome data could not be extracted from the paper, (3) duplicate literature, (4) the full-text of the literature is not available, (5) clinical study on the treatment of DPN complications (such as foot ulcer), (6) DPN with other symptoms (such as cerebrovascular disease), (7) repeated publications, and (8) nonclinical studies.

### 2.3. Study Selection and Data Extraction

The retrieved publications were imported into EndNote, and duplicates were removed after an independent review by two reviewers. Two additional reviewers independently read the titles and abstracts of the remaining papers and removed articles that did not meet the requirements, such as reviews and conference papers. Finally, the literature studies were read after the secondary screening in detail. According to the established inclusion and exclusion criteria, eligible literature studies were screened out and included in the final study. If there were any disagreements during this process, a third-party expert was consulted with years of experience in evidence-based medicine.

To ensure the accuracy of the data and the rigor of the research, two reviewers independently extracted relevant data according to established standards. Extracted information included study author and year, baseline, interventions, demographic characteristics, and outcome measures, among others. Reviewers collaborated and examined each other after extracting and perfecting the data.

### 2.4. Quality Assessment

The revised Cochrane randomized trials risk of bias tool (RoB 2.0) [17] was used to assess the risk of bias for each included study. The following aspects were assessed: randomization process, deviation from intended interventions, incomplete outcome data, bias in outcome measures, and selective reporting. Based on these domains, the overall the risk of bias for each included study was assessed. Two authors independently assessed the risk of bias for each study and cross-validated. If there was disagreement over the attribution of bias, it was resolved through discussion and consensus with a third author.

### 2.5. Data Synthesis and Statistical Analysis

We used the “robvis” package (version 0.3.0) to assess the risk of bias for the included studies and output a risk of bias map. The outcome indicators were continuous data, the effect size was the mean difference (MD), and the 95% confidence interval (CrI) of the effect size was calculated. In the absence of mean and standard deviation, we used the equations published by Wan et al. [18]. We converted the median and interquartile range from the original literature to mean and standard deviation.

We used the “gemtc” package (version 1.0-0) [19] for network meta-analysis. This package utilizes the “rjags” package (versions 4–10) [20] to call Just Another Gibbs Sampler (JAGS version 4.3.0) to perform network meta-analysis in a Bayesian framework. A random-effects model (RE) and a fixed-effects model (FE) were constructed for each outcome indicator, and an appropriate analytical model was selected based on *I*^2^, deviation information criterion (DIC), and average posterior residual deviance. The unknown parameters in the model are estimated using the MCMC (Monte Carlo Markov chain) method. Each model has four Markov chains, each with 100,000 iterations and 20,000 adaptation (or tuning) iterations. The network evidence graph can fully display the evidence characteristics. Among them, the thickness of the line represents the number of studies of the treatment, and the size of the dots represents the total sample size of the intervention; the line between the dots indicates that there is evidence to directly compare the two interventions, and no line indicates that there is no direct comparison between the original studies. At the same time, the surface under the cumulative ordination curve (SUCRA) value for each intervention was calculated and the probability ranking graph was plotted. Judgment and interpretation of the effects of interventions were performed by comparing SUCRA values and probability rankings. The SUCRA value ranges from 0% to 100%; the closer it is to 100%, the better the curative effect, and the closer to 0%, the worse the curative effect [21]. Heterogeneity of treatment effects between studies was assessed using the posterior distribution *τ*^2^ and *I*^2^ statistics. The significance level of the heterogeneity test was *α* = 0.10, and the magnitude of heterogeneity was quantitatively determined by *I*^2^. The method of node splitting is used to test inconsistency. If the *p* value is less than 0.05, there is an inconsistency between direct comparison and indirect comparison and further analysis of the source of inconsistency is required [22, 23].

All the studies were performed in *R* (version 4.1.0) and RStudio (version 1.4.1717-3).

## 3. Results

### 3.1. Identified Publications

**Figure 1:**
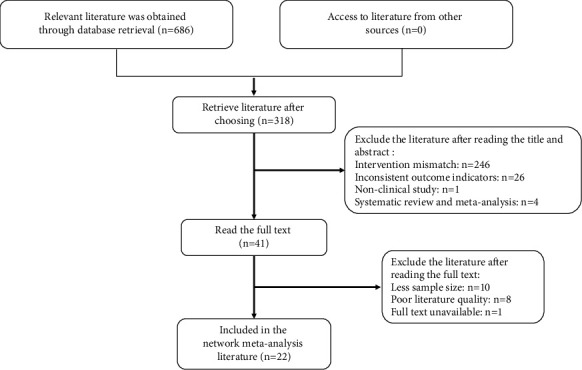
Literature screening process.

A total of 686 articles were retrieved. The bibliographies retrieved from various databases were imported into EndNote software, and 318 references were obtained by using the same software for rechecking. After screening, a total of 22 articles were finally included in RCT. The literature screening process is shown in Figure 1. 22 publications were included. Across the 22 studies, the average age of most study groups was between 50 and 70 years; the proportions of male and female patients were similar. Detailed information on the literature is found in Supplementary Table S1.

**Figure 2:**
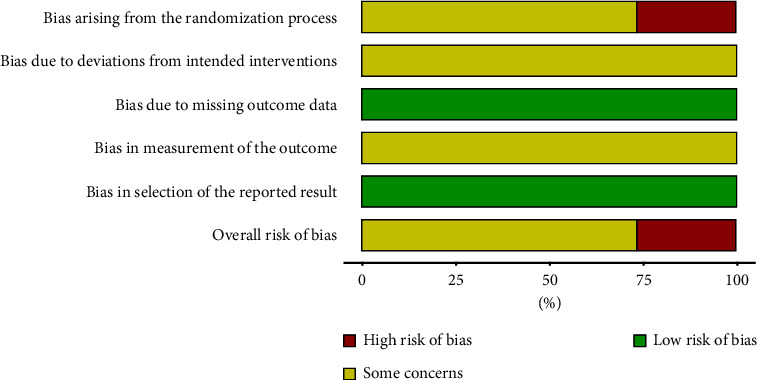
Literature bias risk chart.

17 publications were judged to be unclear, and 5 publications had a high-risk bias. The evaluation criteria were as follows: 5 articles did not mention the specific random method, and none of the 22 publications mentioned blindness and distribution concealment. Since it is difficult to achieve blindness in TCM external treatments, the publications that did not mention blindness were judged as unclear. There was no mention of case shedding, withdrawal, or loss of follow-up in the 22 publications included, and the data were complete (Figure 2, Supplementary Table S2).

**Figure 3:**
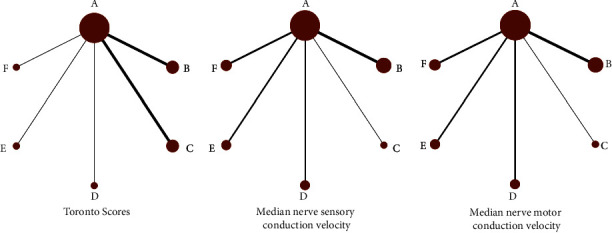
Evidence networks for different outcome indicators compared to various TCM external treatment methods. Treatments of the various groups are as follows: A represents the group receiving mecobalamin treatment, B represents the group receiving acupuncture combined with mecobalamin treatment, C represents the group receiving TCM foot bath combined with mecobalamin treatment, D represents the group receiving acupoint application combined with mecobalamin treatment, E represents the group receiving acupoint injection combined with mecobalamin treatment, and F represents the group receiving TCM fumigation combined with mecobalamin treatment.

Among the 22 articles included, the TCM external treatment methods used were acupuncture, TCM foot bath, acupoint application, acupoint injection, and TCM fumigation. The network evidence analysis of the outcome indicators (Toronto score, median nerve sensory conduction velocity, and median nerve motor conduction velocity) of various TCM external treatment methods found that (Figure 3) five TCM external treatment methods were not compared with each other and only mecobalamin was used as the control group. Therefore, the evidence networks for the outcome indicators were all two-arm studies.

### 3.2. Network Meta-Analysis Results

#### 3.2.1. Toronto Score

**Figure 4:**
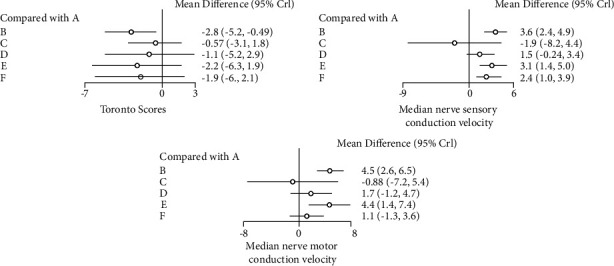
Forest map meta-analysis of different outcome indicators. Treatments of the various groups are as follows: A represents the group receiving mecobalamin treatment, B represents the group receiving acupuncture combined with mecobalamin treatment, C represents the group receiving TCM foot bath combined with mecobalamin treatment, D represents the group receiving acupoint application combined with mecobalamin treatment, E represents the group receiving acupoint injection combined with mecobalamin treatment, and F represents the group receiving TCM fumigation combined with mecobalamin treatment.

**Figure 5:**
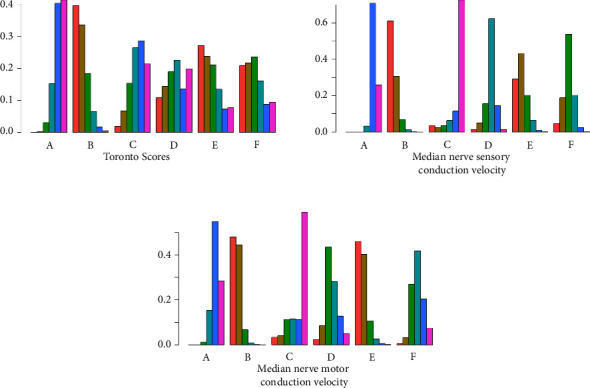
Meta-analysis probability rankings for different interventions and outcomes. Treatments of the various groups are as follows: A represents the group receiving mecobalamin treatment, B represents the group receiving acupuncture combined with mecobalamin treatment, C represents the group receiving TCM foot bath combined with mecobalamin treatment, D represents the group receiving acupoint application combined with mecobalamin treatment, E represents the group receiving acupoint injection combined with mecobalamin treatment, and F represents the group receiving TCM fumigation combined with mecobalamin treatment.

In terms of Toronto score, the use of acupuncture combined with mecobalamin significantly decreased the Toronto score in patients with DPN compared with mecobalamin alone (MD = −2.8, 95% CI: −5.2∼−0.49) (Figure 4). SUCRA showed that the best treatment in terms of decreasing the Toronto score was acupuncture combined with mecobalamin (80%), followed by acupoint injection combined with mecobalamin (66%), and the third was TCM fumigation combined with mecobalamin (60%) (Table 1). The results in Figure 5 show that the ranking of TCM external treatment methods to decrease the Toronto score is acupuncture combined with mecobalamin (B) > acupoint injection combined with mecobalamin (E) > TCM fumigation combined with mecobalamin (F) > acupoint application combined with mecobalamin (D) > TCM foot bath combined with mecobalamin (C) > mecobalamin. This suggests that acupuncture decreases the Toronto score the most. The specific results of the comparison of the effect of five different TCM external treatment methods combined with mecobalamin on decreasing the Toronto score are shown in Supplementary Materials (Table S3).

### 3.3. Median Neural Analysis

#### 3.3.1. Median Nerve Sensory Conduction Velocity

As shown in Figure 4, compared with mecobalamin alone, acupuncture combined with mecobalamin, acupoint injection combined with mecobalamin, and TCM fumigation combined with mecobalamin significantly improved the median nerve sensory conduction velocity in patients with DPN. The median nerve sensory conduction velocity increased the most in the acupuncture combined with mecobalamin group (group B) (MD = 3.6, 95% CI: 2.4∼4.9), followed by acupoint injection combined with mecobalamin (group E) (MD = 3.1, 95% CI: 1.4∼5.0) and TCM fumigation combined with mecobalamin (group F) (MD = 2.4, 95% CI: 1.0∼3.9). SUCRA showed that the best treatment for improving median nerve sensory conduction velocity was acupuncture combined with mecobalamin (90%), followed by acupoint injection combined with mecobalamin (79%), and the third was TCM fumigation combined with mecobalamin (61%) (Table 1). The results in Figure 5 show that the order of improving median nerve sensory conduction velocity by external treatment of traditional Chinese medicine is acupuncture combined with mecobalamin (B) > acupoint injection combined with mecobalamin (E) > TCM fumigation combined with mecobalamin (F) > acupoint application combined with mecobalamin (D) > mecobalamin > TCM foot bath combined with mecobalamin (C). This suggests that acupuncture can best improve the median nerve sensory conduction velocity. The comparison of the effect of five different TCM external treatment methods combined with mecobalamin on the improvement of median nerve sensory conduction velocity is shown in Supplementary Materials (Table S4).

#### 3.3.2. Median Nerve Motor Conduction Velocity

As shown in Figure 4, compared with mecobalamin alone, acupuncture combined with mecobalamin and acupoint injection combined with mecobalamin significantly improved the median nerve motor conduction velocity in patients with DPN. The median nerve motor conduction velocity increased the most in acupuncture combined with mecobalamin (group B) (MD = 4.5, 95% CI: 2.6∼6.5), followed by acupoint injection combined with mecobalamin (group E) (MD = 4.4, 95% CI: 1.4∼7.4). SUCRA showed that the best treatment for improving the median nerve motor conduction velocity was acupuncture combined with mecobalamin (88%), followed by acupoint injection combined with mecobalamin (86%), and the third was acupoint application combined with mecobalamin (49%) (Table 1). The results in Figure 5 show that the order of improving the median nerve motor conduction velocity by external treatment of traditional Chinese medicine is acupuncture combined with mecobalamin (B) > acupoint injection combined with mecobalamin (E) > acupoint application combined with mecobalamin (D) > TCM fumigation combined with mecobalamin (F) > mecobalamin > TCM foot bath combined with mecobalamin (C). This suggests that acupuncture can best improve the median nerve motor conduction velocity. The comparison of the effect of five different TCM external treatment methods combined with mecobalamin on the improvement of median nerve motor conduction velocity is shown in Supplementary Materials (Table S5).

### 3.4. Network Heterogeneity and Inconsistency

To select an appropriate model for meta-analysis, we constructed random- and fixed-effects models for each outcome indicator. The results showed that for all outcome indicators, the *I*^2^ of the random-effects model was smaller than that of the fixed-effects model. Meanwhile, both the DIC and the mean posterior residuals of the random-effects model were smaller than those of the fixed-effects model (Supplementary Table S6). For all outcome indicators, we used random-effects models for network meta-analysis. Assessment of global heterogeneity found *I*^2^ > 60% for all indicators, indicating high global heterogeneity (Supplementary Table S7). All studies were 2-arm studies with mecobalamin alone as a control, with no indirect comparisons and only network comparisons. Therefore, the inconsistency check cannot be performed by the node splitting method. The convergence of the model was analyzed, and it was found that each MCMC chain converged to around 1 within 2000 iterations and was relatively stable. Trace plots, density plots, and convergence diagnostic plots for each outcome indicator are shown in Supplementary Materials (Figures S1–S3).

## 4. Discussion

This study searched Chinese and English databases through corresponding search strategies, and a total of 22 randomized controlled trials were selected, all of which were Chinese publications, with a total of 1885 subjects. The Toronto score and median nerve conduction velocity were statistically analyzed in each study.

The Toronto score is a comprehensive scoring system based on the clinical characteristics of DPN. The clinical outcomes of DPN are recorded and compared comprehensively to reflect physiological and pathological changes with high accuracy [24, 25]. The score is composed of three parts, including sensory function, nerve reflex, and neurological symptom score, and is highly consistent with sural nerve electrophysiological and neuromorphological examination [26]. In this study, there was heterogeneity among the publications, with the Toronto score as the outcome indicator (Figure 4), indicating differences in case sources and administration methods among the studies, so a random-effects model was adopted. For decreasing the Toronto score, acupuncture combined with mecobalamin showed the greatest therapeutic effect, followed by acupoint injection combined with mecobalamin (Figure 4).

In addition to the evaluation of clinical symptoms of DPN patients, the objective and quantitative electromyogram is an important therapeutic index for DPN, which can evaluate the condition of large fiber neuropathy. Studies have shown that neuro-electrophysiological methods can detect peripheral nerve damage earlier and better than clinical methods [27]. Guidelines recommend neuro-electrophysiological examination to assess neurological function in patients with asymptomatic DPN [1]. Our study shows that for improving median nerve sensory/motor conduction velocity, acupuncture combined with mecobalamin showed the greatest therapeutic effect, followed by acupoint injection combined with mecobalamin. In conclusion, our assessment overall found that acupuncture combined with mecobalamin and acupoint injection combined with mecobalamin may be the most effective TCM external treatment methods.

Modern physicians summarize the pathogenesis of DPN mainly according to the following aspects: qi deficiency, qi and yin deficiency, qi deficiency and blood stasis, spleen and kidney deficiency, phlegm and blood stasis, and collateral obstruction [28]. Acupuncture stimulates local acupoints through the application of acupoint selection and tonifying and reducing techniques, which is beneficial to qi and blood circulation and fits the pathogenesis of DPN. Modern medical studies have shown that DPN local acupuncture can significantly increase the pain threshold, effectively improve peripheral nerve microcirculation, eliminate local inflammation, promote edema regression, accelerate the absorption of local degeneration and necrotic substances, and thus promote nerve myelin sheath and axon regeneration [29]. Clinical RCT studies showed that the amplitude of the sural nerve in DPN patients was significantly improved after acupuncture treatment, indicating that acupuncture stimulates nerve regeneration [30]. The results of this study also showed that acupuncture + mecobalamin altered the clinical symptoms and caused electrophysiological improvement of nerve conduction velocity in DPN patients, compared with the other 4 external treatments. Treating DPN with acupuncture used to stimulate acupoints is a new method in modern Chinese medicine.

Compared with traditional acupuncture and moxibustion, acupoint injection is simpler in acupoint selection and shorter in operation time. After injection, the drug remains in the acupoint for a certain period of time to stimulate the corresponding acupoints, so as to prolong the acupuncture sensation and extend the validity of drug treatment [31]. The mechanism of acupoint injection involved the regulation of nerve conduction, neuron signal pathways, protein expression, and oxidative stress levels [32]. Studies have shown that acupoint injection of vitamin B12 differs from the intramuscular injection of the gluteus maximus muscle in alleviating clinical symptoms and improving nerve transmission speed in DPN patients and acupoint injection is superior to intramuscular injection of the gluteus maximus muscle. Thus, acupoint injection is superior to gluteus maximus intramuscular injection [12, 33].

The results showed that TCM fumigation combined with mecobalamin and acupoint application combined with mecobalamin could decrease the Toronto score and improve the median nerve motor/sensory conduction velocity, and the effect is inferior to acupoint injection combined with mecobalamin but superior to foot bath combined with mecobalamin and mecobalamin alone. TCM fumigation is one of the TCM external treatments. This treatment method fumigates the local skin directly through the drug solution and is absorbed and transmitted through the skin and meridians to stimulate nerves and blood vessels, expand the blood vessels of the extremities, improve the blood circulation and local nerve conduction function, and effectively relieve the clinical symptoms of DPN such as pain, fatigue, and numbness [34]. Studies have shown that TCM fumigation can promote blood circulation, improve tissue oxygen and blood supply, repair damaged nerve cells [35], and inhibit the expression of proinflammatory factors [36] to achieve therapeutic purposes. Acupoint application therapy is to achieve the purpose of treatment through the direct effect of drug absorption in specific sites and the indirect effect of acupuncture point stimulation to stimulate the qi [37]. Acupoints have external sensitivity, storage, and amplification effects on drugs. The nerves at the acupoints are distributed in a network. By stimulating these acupoints, the sympathetic nerve can be excited, the vagus nerve tension can be reduced, the nerve-humoral-immune regulatory system can be activated, and strong pharmacological effects can be produced in the corresponding tissues and organs to achieve the therapeutic effect [38].

Therefore, combined with the Toronto score and network meta-analysis of median nerve motor and sensory conduction velocity, the results show that acupuncture combined with mecobalamin may be the most effective method in DPN treatment. Acupoint injection combined with mecobalamin also plays an important role in the treatment of DPN. Clinically, to improve clinical symptoms and median nerve conduction velocity, the above two methods are preferred.

Based on the above conclusions, the design of this study has three shortcomings (the most important is related to the literature included): ① The publications included were all Chinese, and the blind method was difficult to implement in TCM external therapies. Some studies only mentioned “randomization” but do not elaborate on the implementation process of the randomization method, and most of the studies do not mention the method of allocation hiding, so there are different degrees of selection bias and implementation bias. ② After heterogeneity analysis, the trials included were highly heterogeneous in terms of age, course of the disease, follow-up time, and intervention. ③ All the studies included in this paper were single-center studies, and the follow-up time of some studies was less than 1 month, so there was a lack of multicenter, large-sample, long-termfollow-up randomized controlled studies. Therefore, for the above reasons, the quality of the literature included in this study was low, and this has a certain impact on the quality of data in the study. In addition, in the network meta-analysis, because there is no comparison between TCM external treatments, the evidence network diagram in this study did not form a closed network. In addition, there were differences in the implementation methods, acupoint selection and action time of acupuncture, acupoint injection, and other intervention measures in each study. This study did not perform further subgroup analysis, so the ranking results obtained in this paper serve as a reference only. Multicenters, larger-sample, and high-quality studies are needed to further guide clinical practice.

## 5. Conclusions

Combined with the Toronto score and network meta-analysis of median motor nerve sensory conduction velocity, the results show that acupuncture combined with mecobalamin may be the most effective method in DPN treatment. Acupoint injection combined with mecobalamin also plays an important role in the treatment of DPN. Clinically, to improve clinical symptoms and median nerve conduction velocity, the above two methods are preferred.

## Figures and Tables

**Table 1:** SUCRA values of different interventions and outcomes were analyzed.

	A	B	C	D	E	F
Toronto score	0.16	0.80	0.33	0.45	0.66	0.60
Median nerve sensory conduction velocity	0.16	0.90	0.13	0.43	0.79	0.61
Median nerve motor conduction velocity	0.19	0.88	0.20	0.49	0.86	0.40

Treatments of the various groups are as follows: A represents the group receiving mecobalamin treatment, B represents the group receiving acupuncture combined with mecobalamin treatment, C represents the group receiving TCM foot bath combined with mecobalamin treatment, D represents the group receiving acupoint application combined with mecobalamin treatment, E represents the group receiving acupoint injection combined with mecobalamin treatment, and F represents the group receiving TCM fumigation combined with mecobalamin treatment.

## Data Availability

The data used to support the findings of this study are available from the corresponding author upon request.

## Supplementary Materials

Supplementary Materials Appendix 1: search strategy of PubMed. Table S1: summary of literature information. Table S2: quality evaluation of the studies included. Table S3: results of network meta-analysis for Toronto score. Table S4: results of network meta-analysis of median nerve sensory conduction velocity. Table S5: results of network meta-analysis of median nerve motor conduction velocity. Table S6: model fit statistics for all outcomes. Table S7: heterogeneity assessment in the network. Figure S1: the trace map, density map, and convergent diagnostic diagram of Toronto score. Figure S2: the trace map, density map, and convergent diagnostic diagram of median nerve sensory conduction velocity. Figure S3: the trace map, density map, and convergent diagnostic diagram of median nerve motor conduction velocity.
