# Supplementary material for: Five Traditional Chinese Medicine External Treatment Methods Combined with Mecobalamin for Diabetic Peripheral Neuropathy: A Network Meta-Analysis
Source: Evid Based Complement Alternat Med. 2022 Dec 16;2022:4251022. doi: 10.1155/2022/4251022 (PMC9788892; doi:10.1155/2022/4251022)
Supplement: Supplementary Materials — Appendix 1: search strategy of PubMed. Table S1: summary of literature information. Table S2: quality evaluation of the studies included. Table S3: results of network meta-analysis for Toronto score. Table S4: results of network meta-analysis of median nerve sensory conduction velocity. Table S5: results of network meta-analysis of median nerve motor conduction velocity. Table S6: model fit statistics for all outcomes. Table S7: heterogeneity assessment in the network. Figure S1: the trace map, density map, and convergent diagnostic diagram of Toronto score. Figure S2: the trace map, density map, and convergent diagnostic diagram of median nerve sensory conduction velocity. Figure S3: the trace map, density map, and convergent diagnostic diagram of median nerve motor conduction velocity. [file 4251022.f1.docx]

**Appendix 1: Search strategy for RCTs assessing the effects of 5 external TCM treatments in the treatment of patients with diabetic peripheral neuropathy**

**PubMed**

**① Acupuncture combined with mecobalamine**

(((("Diabetic Neuropathies"[Mesh]) OR (((((((((((((((((((((((((((((((((((((((((((((Diabetic Neuropathy[Title/Abstract]) OR (Neuropathies, Diabetic[Title/Abstract])) OR (Neuropathy, Diabetic[Title/Abstract])) OR (Diabetic Autonomic Neuropathy[Title/Abstract])) OR (Autonomic Neuropathies, Diabetic[Title/Abstract])) OR (Autonomic Neuropathy, Diabetic[Title/Abstract])) OR (Diabetic Autonomic Neuropathies[Title/Abstract])) OR (Neuropathies, Diabetic Autonomic[Title/Abstract])) OR (Neuropathy, Diabetic Autonomic[Title/Abstract])) OR (Diabetic Neuralgia[Title/Abstract])) OR (Diabetic Neuralgias[Title/Abstract])) OR (Neuralgias, Diabetic[Title/Abstract])) OR (Diabetic Neuropathy, Painful[Title/Abstract])) OR (Diabetic Neuropathies, Painful[Title/Abstract])) OR (Neuropathies, Painful Diabetic[Title/Abstract])) OR (Neuropathy, Painful Diabetic[Title/Abstract])) OR (Painful Diabetic Neuropathies[Title/Abstract])) OR (Painful Diabetic Neuropathy[Title/Abstract])) OR (Neuralgia, Diabetic[Title/Abstract])) OR (Symmetric Diabetic Proximal Motor Neuropathy[Title/Abstract])) OR (Asymmetric Diabetic Proximal Motor Neuropathy[Title/Abstract])) OR (Diabetic Asymmetric Polyneuropathy[Title/Abstract])) OR (Asymmetric Polyneuropathies, Diabetic[Title/Abstract])) OR (Asymmetric Polyneuropathy, Diabetic[Title/Abstract])) OR (Diabetic Asymmetric Polyneuropathies[Title/Abstract])) OR (Polyneuropathies, Diabetic Asymmetric[Title/Abstract])) OR (Polyneuropathy, Diabetic Asymmetric[Title/Abstract])) OR (Diabetic Mononeuropathy[Title/Abstract])) OR (Diabetic Mononeuropathies[Title/Abstract])) OR (Mononeuropathies, Diabetic[Title/Abstract])) OR (Mononeuropathy, Diabetic[Title/Abstract])) OR (Diabetic Mononeuropathy Simplex[Title/Abstract])) OR (Diabetic Mononeuropathy Simplices[Title/Abstract])) OR (Mononeuropathy Simplex, Diabetic[Title/Abstract])) OR (Mononeuropathy Simplices, Diabetic[Title/Abstract])) OR (Simplex, Diabetic Mononeuropathy[Title/Abstract])) OR (Simplices, Diabetic Mononeuropathy[Title/Abstract])) OR (Diabetic Amyotrophy[Title/Abstract])) OR (Amyotrophies, Diabetic[Title/Abstract])) OR (Amyotrophy, Diabetic[Title/Abstract])) OR (Diabetic Amyotrophies[Title/Abstract])) OR (Diabetic Polyneuropathy[Title/Abstract])) OR (Diabetic Polyneuropathies[Title/Abstract])) OR (Polyneuropathies, Diabetic[Title/Abstract])) OR (Polyneuropathy, Diabetic[Title/Abstract]))) AND (mecobalamine[Title/Abstract])) AND (randomized controlled trial [Publication Type] OR randomized [Title/Abstract] OR placebo [Title/Abstract])) AND ((Acupuncture[Title/Abstract]) OR (Pharmacopuncture[Title/Abstract]))

**② TCM foot bath combined with mecobalamine**

((("Diabetic Neuropathies"[Mesh]) OR (((((((((((((((((((((((((((((((((((((((((((((Diabetic Neuropathy[Title/Abstract]) OR (Neuropathies, Diabetic[Title/Abstract])) OR (Neuropathy, Diabetic[Title/Abstract])) OR (Diabetic Autonomic Neuropathy[Title/Abstract])) OR (Autonomic Neuropathies, Diabetic[Title/Abstract])) OR (Autonomic Neuropathy, Diabetic[Title/Abstract])) OR (Diabetic Autonomic Neuropathies[Title/Abstract])) OR (Neuropathies, Diabetic Autonomic[Title/Abstract])) OR (Neuropathy, Diabetic Autonomic[Title/Abstract])) OR (Diabetic Neuralgia[Title/Abstract])) OR (Diabetic Neuralgias[Title/Abstract])) OR (Neuralgias, Diabetic[Title/Abstract])) OR (Diabetic Neuropathy, Painful[Title/Abstract])) OR (Diabetic Neuropathies, Painful[Title/Abstract])) OR (Neuropathies, Painful Diabetic[Title/Abstract])) OR (Neuropathy, Painful Diabetic[Title/Abstract])) OR (Painful Diabetic Neuropathies[Title/Abstract])) OR (Painful Diabetic Neuropathy[Title/Abstract])) OR (Neuralgia, Diabetic[Title/Abstract])) OR (Symmetric Diabetic Proximal Motor Neuropathy[Title/Abstract])) OR (Asymmetric Diabetic Proximal Motor Neuropathy[Title/Abstract])) OR (Diabetic Asymmetric Polyneuropathy[Title/Abstract])) OR (Asymmetric Polyneuropathies, Diabetic[Title/Abstract])) OR (Asymmetric Polyneuropathy, Diabetic[Title/Abstract])) OR (Diabetic Asymmetric Polyneuropathies[Title/Abstract])) OR (Polyneuropathies, Diabetic Asymmetric[Title/Abstract])) OR (Polyneuropathy, Diabetic Asymmetric[Title/Abstract])) OR (Diabetic Mononeuropathy[Title/Abstract])) OR (Diabetic Mononeuropathies[Title/Abstract])) OR (Mononeuropathies, Diabetic[Title/Abstract])) OR (Mononeuropathy, Diabetic[Title/Abstract])) OR (Diabetic Mononeuropathy Simplex[Title/Abstract])) OR (Diabetic Mononeuropathy Simplices[Title/Abstract])) OR (Mononeuropathy Simplex, Diabetic[Title/Abstract])) OR (Mononeuropathy Simplices, Diabetic[Title/Abstract])) OR (Simplex, Diabetic Mononeuropathy[Title/Abstract])) OR (Simplices, Diabetic Mononeuropathy[Title/Abstract])) OR (Diabetic Amyotrophy[Title/Abstract])) OR (Amyotrophies, Diabetic[Title/Abstract])) OR (Amyotrophy, Diabetic[Title/Abstract])) OR (Diabetic Amyotrophies[Title/Abstract])) OR (Diabetic Polyneuropathy[Title/Abstract])) OR (Diabetic Polyneuropathies[Title/Abstract])) OR (Polyneuropathies, Diabetic[Title/Abstract])) OR (Polyneuropathy, Diabetic[Title/Abstract]))) AND (mecobalamine[Title/Abstract])) AND (randomized controlled trial [Publication Type] OR randomized [Title/Abstract] OR placebo [Title/Abstract])

**③ Acupoint application combined with mecobalamine**

(((("Diabetic Neuropathies"[Mesh]) OR (((((((((((((((((((((((((((((((((((((((((((((Diabetic Neuropathy[Title/Abstract]) OR (Neuropathies, Diabetic[Title/Abstract])) OR (Neuropathy, Diabetic[Title/Abstract])) OR (Diabetic Autonomic Neuropathy[Title/Abstract])) OR (Autonomic Neuropathies, Diabetic[Title/Abstract])) OR (Autonomic Neuropathy, Diabetic[Title/Abstract])) OR (Diabetic Autonomic Neuropathies[Title/Abstract])) OR (Neuropathies, Diabetic Autonomic[Title/Abstract])) OR (Neuropathy, Diabetic Autonomic[Title/Abstract])) OR (Diabetic Neuralgia[Title/Abstract])) OR (Diabetic Neuralgias[Title/Abstract])) OR (Neuralgias, Diabetic[Title/Abstract])) OR (Diabetic Neuropathy, Painful[Title/Abstract])) OR (Diabetic Neuropathies, Painful[Title/Abstract])) OR (Neuropathies, Painful Diabetic[Title/Abstract])) OR (Neuropathy, Painful Diabetic[Title/Abstract])) OR (Painful Diabetic Neuropathies[Title/Abstract])) OR (Painful Diabetic Neuropathy[Title/Abstract])) OR (Neuralgia, Diabetic[Title/Abstract])) OR (Symmetric Diabetic Proximal Motor Neuropathy[Title/Abstract])) OR (Asymmetric Diabetic Proximal Motor Neuropathy[Title/Abstract])) OR (Diabetic Asymmetric Polyneuropathy[Title/Abstract])) OR (Asymmetric Polyneuropathies, Diabetic[Title/Abstract])) OR (Asymmetric Polyneuropathy, Diabetic[Title/Abstract])) OR (Diabetic Asymmetric Polyneuropathies[Title/Abstract])) OR (Polyneuropathies, Diabetic Asymmetric[Title/Abstract])) OR (Polyneuropathy, Diabetic Asymmetric[Title/Abstract])) OR (Diabetic Mononeuropathy[Title/Abstract])) OR (Diabetic Mononeuropathies[Title/Abstract])) OR (Mononeuropathies, Diabetic[Title/Abstract])) OR (Mononeuropathy, Diabetic[Title/Abstract])) OR (Diabetic Mononeuropathy Simplex[Title/Abstract])) OR (Diabetic Mononeuropathy Simplices[Title/Abstract])) OR (Mononeuropathy Simplex, Diabetic[Title/Abstract])) OR (Mononeuropathy Simplices, Diabetic[Title/Abstract])) OR (Simplex, Diabetic Mononeuropathy[Title/Abstract])) OR (Simplices, Diabetic Mononeuropathy[Title/Abstract])) OR (Diabetic Amyotrophy[Title/Abstract])) OR (Amyotrophies, Diabetic[Title/Abstract])) OR (Amyotrophy, Diabetic[Title/Abstract])) OR (Diabetic Amyotrophies[Title/Abstract])) OR (Diabetic Polyneuropathy[Title/Abstract])) OR (Diabetic Polyneuropathies[Title/Abstract])) OR (Polyneuropathies, Diabetic[Title/Abstract])) OR (Polyneuropathy, Diabetic[Title/Abstract]))) AND (mecobalamine[Title/Abstract])) AND (randomized controlled trial [Publication Type] OR randomized [Title/Abstract] OR placebo [Title/Abstract])) AND (Traditional Chinese medicine sticking[Title/Abstract])

**④ Acupoint injection combined with mecobalamine**

(((("Diabetic Neuropathies"[Mesh]) OR (((((((((((((((((((((((((((((((((((((((((((((Diabetic Neuropathy[Title/Abstract]) OR (Neuropathies, Diabetic[Title/Abstract])) OR (Neuropathy, Diabetic[Title/Abstract])) OR (Diabetic Autonomic Neuropathy[Title/Abstract])) OR (Autonomic Neuropathies, Diabetic[Title/Abstract])) OR (Autonomic Neuropathy, Diabetic[Title/Abstract])) OR (Diabetic Autonomic Neuropathies[Title/Abstract])) OR (Neuropathies, Diabetic Autonomic[Title/Abstract])) OR (Neuropathy, Diabetic Autonomic[Title/Abstract])) OR (Diabetic Neuralgia[Title/Abstract])) OR (Diabetic Neuralgias[Title/Abstract])) OR (Neuralgias, Diabetic[Title/Abstract])) OR (Diabetic Neuropathy, Painful[Title/Abstract])) OR (Diabetic Neuropathies, Painful[Title/Abstract])) OR (Neuropathies, Painful Diabetic[Title/Abstract])) OR (Neuropathy, Painful Diabetic[Title/Abstract])) OR (Painful Diabetic Neuropathies[Title/Abstract])) OR (Painful Diabetic Neuropathy[Title/Abstract])) OR (Neuralgia, Diabetic[Title/Abstract])) OR (Symmetric Diabetic Proximal Motor Neuropathy[Title/Abstract])) OR (Asymmetric Diabetic Proximal Motor Neuropathy[Title/Abstract])) OR (Diabetic Asymmetric Polyneuropathy[Title/Abstract])) OR (Asymmetric Polyneuropathies, Diabetic[Title/Abstract])) OR (Asymmetric Polyneuropathy, Diabetic[Title/Abstract])) OR (Diabetic Asymmetric Polyneuropathies[Title/Abstract])) OR (Polyneuropathies, Diabetic Asymmetric[Title/Abstract])) OR (Polyneuropathy, Diabetic Asymmetric[Title/Abstract])) OR (Diabetic Mononeuropathy[Title/Abstract])) OR (Diabetic Mononeuropathies[Title/Abstract])) OR (Mononeuropathies, Diabetic[Title/Abstract])) OR (Mononeuropathy, Diabetic[Title/Abstract])) OR (Diabetic Mononeuropathy Simplex[Title/Abstract])) OR (Diabetic Mononeuropathy Simplices[Title/Abstract])) OR (Mononeuropathy Simplex, Diabetic[Title/Abstract])) OR (Mononeuropathy Simplices, Diabetic[Title/Abstract])) OR (Simplex, Diabetic Mononeuropathy[Title/Abstract])) OR (Simplices, Diabetic Mononeuropathy[Title/Abstract])) OR (Diabetic Amyotrophy[Title/Abstract])) OR (Amyotrophies, Diabetic[Title/Abstract])) OR (Amyotrophy, Diabetic[Title/Abstract])) OR (Diabetic Amyotrophies[Title/Abstract])) OR (Diabetic Polyneuropathy[Title/Abstract])) OR (Diabetic Polyneuropathies[Title/Abstract])) OR (Polyneuropathies, Diabetic[Title/Abstract])) OR (Polyneuropathy, Diabetic[Title/Abstract]))) AND (mecobalamine[Title/Abstract])) AND (randomized controlled trial [Publication Type] OR randomized [Title/Abstract] OR placebo [Title/Abstract])) AND (Point injection[Title/Abstract])

**⑤TCM fumigation combined with mecobalamine**

(((("Diabetic Neuropathies"[Mesh]) OR (((((((((((((((((((((((((((((((((((((((((((((Diabetic Neuropathy[Title/Abstract]) OR (Neuropathies, Diabetic[Title/Abstract])) OR (Neuropathy, Diabetic[Title/Abstract])) OR (Diabetic Autonomic Neuropathy[Title/Abstract])) OR (Autonomic Neuropathies, Diabetic[Title/Abstract])) OR (Autonomic Neuropathy, Diabetic[Title/Abstract])) OR (Diabetic Autonomic Neuropathies[Title/Abstract])) OR (Neuropathies, Diabetic Autonomic[Title/Abstract])) OR (Neuropathy, Diabetic Autonomic[Title/Abstract])) OR (Diabetic Neuralgia[Title/Abstract])) OR (Diabetic Neuralgias[Title/Abstract])) OR (Neuralgias, Diabetic[Title/Abstract])) OR (Diabetic Neuropathy, Painful[Title/Abstract])) OR (Diabetic Neuropathies, Painful[Title/Abstract])) OR (Neuropathies, Painful Diabetic[Title/Abstract])) OR (Neuropathy, Painful Diabetic[Title/Abstract])) OR (Painful Diabetic Neuropathies[Title/Abstract])) OR (Painful Diabetic Neuropathy[Title/Abstract])) OR (Neuralgia, Diabetic[Title/Abstract])) OR (Symmetric Diabetic Proximal Motor Neuropathy[Title/Abstract])) OR (Asymmetric Diabetic Proximal Motor Neuropathy[Title/Abstract])) OR (Diabetic Asymmetric Polyneuropathy[Title/Abstract])) OR (Asymmetric Polyneuropathies, Diabetic[Title/Abstract])) OR (Asymmetric Polyneuropathy, Diabetic[Title/Abstract])) OR (Diabetic Asymmetric Polyneuropathies[Title/Abstract])) OR (Polyneuropathies, Diabetic Asymmetric[Title/Abstract])) OR (Polyneuropathy, Diabetic Asymmetric[Title/Abstract])) OR (Diabetic Mononeuropathy[Title/Abstract])) OR (Diabetic Mononeuropathies[Title/Abstract])) OR (Mononeuropathies, Diabetic[Title/Abstract])) OR (Mononeuropathy, Diabetic[Title/Abstract])) OR (Diabetic Mononeuropathy Simplex[Title/Abstract])) OR (Diabetic Mononeuropathy Simplices[Title/Abstract])) OR (Mononeuropathy Simplex, Diabetic[Title/Abstract])) OR (Mononeuropathy Simplices, Diabetic[Title/Abstract])) OR (Simplex, Diabetic Mononeuropathy[Title/Abstract])) OR (Simplices, Diabetic Mononeuropathy[Title/Abstract])) OR (Diabetic Amyotrophy[Title/Abstract])) OR (Amyotrophies, Diabetic[Title/Abstract])) OR (Amyotrophy, Diabetic[Title/Abstract])) OR (Diabetic Amyotrophies[Title/Abstract])) OR (Diabetic Polyneuropathy[Title/Abstract])) OR (Diabetic Polyneuropathies[Title/Abstract])) OR (Polyneuropathies, Diabetic[Title/Abstract])) OR (Polyneuropathy, Diabetic[Title/Abstract]))) AND (fumigation[Title/Abstract])) AND (mecobalamine[Title/Abstract])) AND (randomized controlled trial [Publication Type] OR randomized [Title/Abstract] OR placebo [Title/Abstract])

| Table S1 Summary of literature information | | | | | | | | | | | | | | | | | |
| --- | --- | --- | --- | --- | --- | --- | --- | --- | --- | --- | --- | --- | --- | --- | --- | --- | --- |
| NO. | **Study** | | | **Sample cases** | **Experimental group** | | | | |  | **Control group** | | | | | **Outcome indicators** | **follow-up time(day)** |
|  |  |  |  |  | **Sample cases** | **M/F** | **Age** | **course of DPN** | **intervention** |  | **Sample cases** | **M/F** | **Age** | **course of**  **DPN** | **intervention** |  |  |
| 1 | WangD2018^[1]^ | 60 | | | 30 | 16/14 | 56.73±8.08 | 12.63±6.11 | C+A（po.） |  | 30 | 17/13 | 56.63±8.56 | 11.87±6.13 | A（po.）500ug tid | ③ | 56 |
| 2 | ZhangY2014^[2]^ | 65 | | | 33 | 16/17 | 62.9±6.7 | 3.22±2.00 | C+A（po.） |  | 32 | 17/15 | 59.9±6.7 | 3.37±2.62 | A（po.）500ug tid | ①②③ | 84 |
| 3 | DuMZ2019^[3]^ | 80 | | | 40 | 27/13 | 54.74±8.19 | 1.59±1.17 | B+A（po.） |  | 40 | 23/17 | 54.67±7.03 | 1.50 ±1. 02 | A（po.）500ug tid | ①③ | 30 |
| 4 | ChenGL2021^[4]^ | 104 | | | 52 | 25/26 | 59.13±1.72 | 4.22±0.83 | B+A（po.） |  | 52 | 28/23 | 58.86±2.05 | 4.09±0.94 | A（po.）500ug tid | ①② | 28 |
| 5 | YangQJ2014^[5]^ | 170 | | | 84 | 48/36 | 59.1±3.6 | - | E+A（po.） |  | 86 | 51/35 | 61.7±4.2 | - | A（po.）500ug tid | ①② | 40 |
| 6 | XuXM2017^[6]^ | 60 | | | 30 | 18/12 | 59.1 | 3.8 | D+A（po.） |  | 30 | 16/14 | 57.6 | 3.5 | A（po.）500ug tid | ①② | 28 |
| 7 | HanLY2016^[7]^ | 66 | | | 33 | 18/15 | 63.1±6.2 | 3.06±0.85 | B+A（po.） |  | 33 | 17/16 | 63.8±5.9 | 2.95±0.89 | A（po.）500ug tid | ①② | 60 |
| 8 | WangJB2018^[8]^ | 90 | | | 45 | 25/20 | 69.7±6.8 | - | B+A（po.） |  | 45 | 26/19 | 70.3±6.1 | - | A（po.）500ug tid | ①② | 21 |
| 9 | WangZ2018^[9]^ | 157 | | | 79 | 42/37 | 63.5±6.9 | 2.1±0.4 | B+A（po.） |  | 78 | 40/38 | 64.7±7.2 | 1.9±0.3 | A（po.）500ug tid | ①② | 56 |
| 10 | WuFH2017^[10]^ | 80 | | | 40 | 13/27 | 53.41±8.31 | 1.79±1.25 | B+A（po.） |  | 40 | 15/25 | 52.97±8.45 | 1.76±1.31 | A（po.）500ug tid | ①②③ | 14 |
| 11 | ChenF2020^[11]^ | 88 | | | 44 | 24/20 | 52.64±5.09 | 6.27±1.19 | E+A（po.） |  | 44 | 23/21 | 53.09±5.17 | 6.24 ±1.41 | A（po.）500ug tid | ①②③ | 84 |
| 12 | ZhouY2015^[12]^ | 94 | | | 48 | 23/25 | 48.9±3.6 | - | F+A（po.） |  | 46 | 26/20 | 50.1±4.3 | - | A（iv.）1000ug qd | ①② | 28 |
| 13 | LiuL2019^[13]^ | 100 | | | 50 | 24/26 | 57.44±12.30 | - | E+A（po.） |  | 50 | 25/25 | 58.03±12.78 | - | A（po.）500ug tid | ①② | 56 |
| 14 | JinZ2020^[14]^ | 60 | | | 30 | 18/12 | 62.18±6.10 | - | B+A（im.） |  | 30 | 20/10 | 61.87±5.92 | - | A（im.）0.5mg qod | ①②③ | 84 |
| 15 | ZhouY2018^[15]^ | 68 | | | 34 | 18/16 | 48.7±3.4 | - | D+A（iv gtt.） |  | 34 | 15/19 | 49.9±4.3 | - | A（iv gtt.）1mg qd | ①②③ | 42 |
| 16 | WangKG2015^[16]^ | | 80 | | 40 | 22/18 | 51.2 | 8.9 | D+A（im.） |  | 40 | 21/19 | 51.9 | 9.2 | A（im.）0.5mg qd | ①② | 14 |
| 17 | WangZ2018^[17]^ | 131 | | | 65 | - | - | - | F+A（po.） |  | 66 | - | - | - | A（po.）0.5mg tid | ①② | 56 |
| 18 | GaoMS2011^[18]^ | 84 | | | 42 | 22/20 | 64.5 | - | F+A（iv.） |  | 42 | 21/21 | 63.5 | - | A（iv.）0.5mg qd | ① | 28 |
| 19 | ZengDY2017^[19]^ | 60 | | | 30 | 18/12 | 60 | - | F+A（po.） |  | 30 | 19/11 | 59.5 | - | A（po.）0.5mg tid | ①② | 28 |
| 20 | LiuF2017^[20]^ | 60 | | | 30 | 14/16 | 51.9±7.9 | 4.67±1.18 | C+A（im.） |  | 30 | 15/15 | 53.23±7.87 | 4.23±1.56 | A（im.）0.5mg qd | ③ | 14 |
| 21 | HanQ2018^[21]^ | 64 | | | 34 | 22/12 | 66.9±3.6 | - | B+A（acupoint injection） |  | 30 | 19/11 | 65.6±4.7 | - | A（acupoint injection）0.5mg qd | ②③ | 28 |
| 22 | WuY2011^[22]^ | 64 | | | 32 | 18/14 | 56.32±7.43 | 2.42±1.37 | C+A（po.） |  | 32 | 16/16 | 56.46±7.23 | 2.47±1.27 | A（po.）0.5mg tid | ①③ | 56 |

**Note: A= mecobalamine, B= acupuncture + mecobalamine, C= TCM foot bath + mecobalamine, D= acupoint application + mecobalamine, E= acupoint injection + mecobalamine, F= TCM fumigation + mecobalamine;** **Outcome indicators: ①Median nerve sensory conduction velocity, ②median nerve motor conduction velocity, and ③Toronto score.**

| Table S2 Quality evaluation of the studies included | | | | | | |
| --- | --- | --- | --- | --- | --- | --- |
| Study | **Randomization process** | **Deviation from intended intervention** | **Missing outcome data** | **Measurement of the outcome** | **Selection of the reported result** | **Overall risk of bias** |
|  |  |  |  |  |  |  |
| WangD2018^[1]^ | Unclear | Unclear | Low | Unclear | Low | Unclear |
| ZhangY2014^[2]^ | Unclear | Unclear | Low | Unclear | Low | Unclear |
| DuMZ2019^[3]^ | Unclear | Unclear | Low | Unclear | Low | Unclear |
| ChenGL2021^[4]^ | Unclear | Unclear | Low | Unclear | Low | Unclear |
| YangQJ2014^[5]^ | Unclear | Unclear | Low | Unclear | Low | Unclear |
| XuXM2017^[6]^ | High | Unclear | Low | Unclear | Low | High |
| HanLY2016^[7]^ | High | Unclear | Low | Unclear | Low | High |
| WangJB2018^[8]^ | High | Unclear | Low | Unclear | Low | High |
| WangZ2018^[9]^ | High | Unclear | Low | Unclear | Low | High |
| WuFH2017^[10]^ | Unclear | Unclear | Low | Unclear | Low | Unclear |
| ChenF2020^[11]^ | Unclear | Unclear | Low | Unclear | Low | Unclear |
| ZhouY2015^[12]^ | Unclear | Unclear | Low | Unclear | Low | Unclear |
| LiuL2019^[13]^ | Unclear | Unclear | Low | Unclear | Low | Unclear |
| JinZ2020^[14]^ | Unclear | Unclear | Low | Unclear | Low | Unclear |
| ZhouY2018^[15]^ | Unclear | Unclear | Low | Unclear | Low | Unclear |
| WangKG2015^[16]^ | Unclear | Unclear | Low | Unclear | Low | Unclear |
| WangZ2018^[17]^ | High | Unclear | Low | Unclear | Low | High |
| GaoMS2011^[18]^ | Unclear | Unclear | Low | Unclear | Low | Unclear |
| ZengDY2017^[19]^ | Unclear | Unclear | Low | Unclear | Low | Unclear |
| LiuF2017^[20]^ | Unclear | Unclear | Low | Unclear | Low | Unclear |
| HanQ2018^[21]^ | Unclear | Unclear | Low | Unclear | Low | Unclear |
| WuY2011^[22]^ | Unclear | Unclear | Low | Unclear | Low | Unclear |

| Table S3 Results of network meta-analysis for Toronto Score | | | | | |
| --- | --- | --- | --- | --- | --- |
| Mecobalamine (A) |  |  |  |  |  |
| 2.82 (0.50, 5.20) ^a^ | **Acupuncture + Mecobalamine (B)** |  |  |  |  |
| 0.57 (-1.76, 3.12) | -2.26 (-5.54, 1.25) | **TCM foot bath + Mecobalamine (C)** |  |  |  |
| 1.12 (-3.00, 5.15) | -1.69 (-6.50, 2.95) | 0.56 (-4.36, 5.17) | **Acupoint application + Mecobalamine (D)** |  |  |
| 2.22 (-1.80, 6.26) | -0.60 (-5.30, 3.99) | 1.65 (-3.17, 6.21) | 1.09 (-4.61, 6.85) | **Acupoint injection + Mecobalamine (E)** |  |
| 1.94 (-2.06, 5.93) | -0.90 (-5.53, 3.72) | 1.37 (-3.37, 5.93) | 0.81 (-4.85, 6.57) | -0.28 (-5.97, 5.39) | **TCM fumigation + Mecobalamine (G)** |

**The reported results are displayed with effect size and 95% confidence interval. Mean difference (MD) is applied to continuous results. a P < 0.05, with statistical significance.**

| Table S4 Results of network meta-analysis of median nerve sensory conduction velocity | | | | | |
| --- | --- | --- | --- | --- | --- |
| Mecobalamine (A) |  |  |  |  |  |
| -3.62 (-4.91, -2.39) ^a^ | **Acupuncture + Mecobalamine (B)** |  |  |  |  |
| 1.95 (-4.36, 8.33) | 5.59 (-0.81, 12.13) | **TCM foot bath + Mecobalamine (C)** |  |  |  |
| -1.48 (-3.45,0.23) | 2.14 (-0.15, 4.25) | -3.46 (-10.12, 3.05) | **Acupoint application + Mecobalamine (D)** |  |  |
| -3.13 (-5.00, -1.34) ^a^ | 0.49 (-1.72, 2.72) | -5.08 (-11.78, 1.45) | -1.65 (-4.17, 1.01) | **Acupoint injection + Mecobalamine (E)** |  |
| -2.38 (-3.88, -1.02) ^a^ | 1.24 (-0.70, 3.11) | -4.34 (-10.91, 2.03) | -0.92 (-3.15, 1.49) | 0.74 (-1.57, 3.07) | **TCM fumigation + Mecobalamine (G)** |

**The reported results are displayed with effect size and 95% confidence interval. Mean difference (MD) is applied to continuous results. a P < 0.05, with statistical significance.**

| Table S5 Results of network meta-analysis of median nerve motor conduction velocity | | | | | |
| --- | --- | --- | --- | --- | --- |
| Mecobalamine (A) |  |  |  |  |  |
| -4.49 (-6.48, -2.52) ^a^ | **Acupuncture + Mecobalamine (B)** |  |  |  |  |
| 0.87 (-5.44, 7.09) | 5.35 (-1.23, 11.96) | **TCM foot bath + Mecobalamine (C)** |  |  |  |
| -1.71 (-4.74,1.22) | 2.78 (-0.84, 6.33) | -2.59 (-9.58, 4.31) | **Acupoint application + Mecobalamine (D)** |  |  |
| -4.40 (-7.37, -1.42) ^a^ | 0.10 (-3.45, 3.69) | -5.25 (-12.22, 1.70) | -2.68 (-6.82, 1.60) | **Acupoint injection + Mecobalamine (E)** |  |
| -1.15 (-3.62,1.29) | 3.34 (0.21, 6.50) | -2.02 (-8.72, 4.73) | 0.55 (-3.26, 4.43) | 3.25 (-0.59, 7.05) | **TCM fumigation + Mecobalamine (G)** |

**The reported results are displayed with effect size and 95% confidence interval. Mean difference (MD) is applied to continuous results. a P < 0.05, with statistical significance.**

| Table S6 Model fit statistics for all outcomes | | | | | | | |
| --- | --- | --- | --- | --- | --- | --- | --- |
| Outcome | **Model** | **DIC** | **Dbar** | **pD** | **ratio** | **I^2** | **Used in base case analyses** |
| Toronto score | FE | 53.64 | 39.59 | 14.05 | 2.199 | 57% | No |
|  | RE | 35.63 | 18.06 | 17.57 | 1.003 | 6% | Yes |
| median nerve sensory conduction velocity | FE | 79.29 | 56.26 | 23.03 | 1.563 | 38% | No |
|  | RE | 70.68 | 38.99 | 31.27 | 1.083 | 10% | Yes |
| median nerve motor conduction velocity | FE | 151.49 | 128.44 | 23.05 | 3.568 | 73% | No |
|  | RE | 70.21 | 35.90 | 34.31 | 0.997 | 3% | Yes |
| DIC, deviance information criterion; FE, fixed effects; RE, random effects. | | | | | | | |
|  | | | | | | | |

| Table S7 Heterogeneity assessment in network | | | | |
| --- | --- | --- | --- | --- |
| Outcomes | **numbers of trails** | | **number of participants** | **heterogeneity(I^2^)** |
| Toronto score | | 9 | 630 | 87.34% |
| median nerve sensory conduction velocity | | 18 | 1638 | 62.93% |
| median nerve motor conduction velocity | | 18 | 1602 | 88.21% |

**Fig S1 The trace map, density map and convergent diagnostic diagram of Toronto Score**


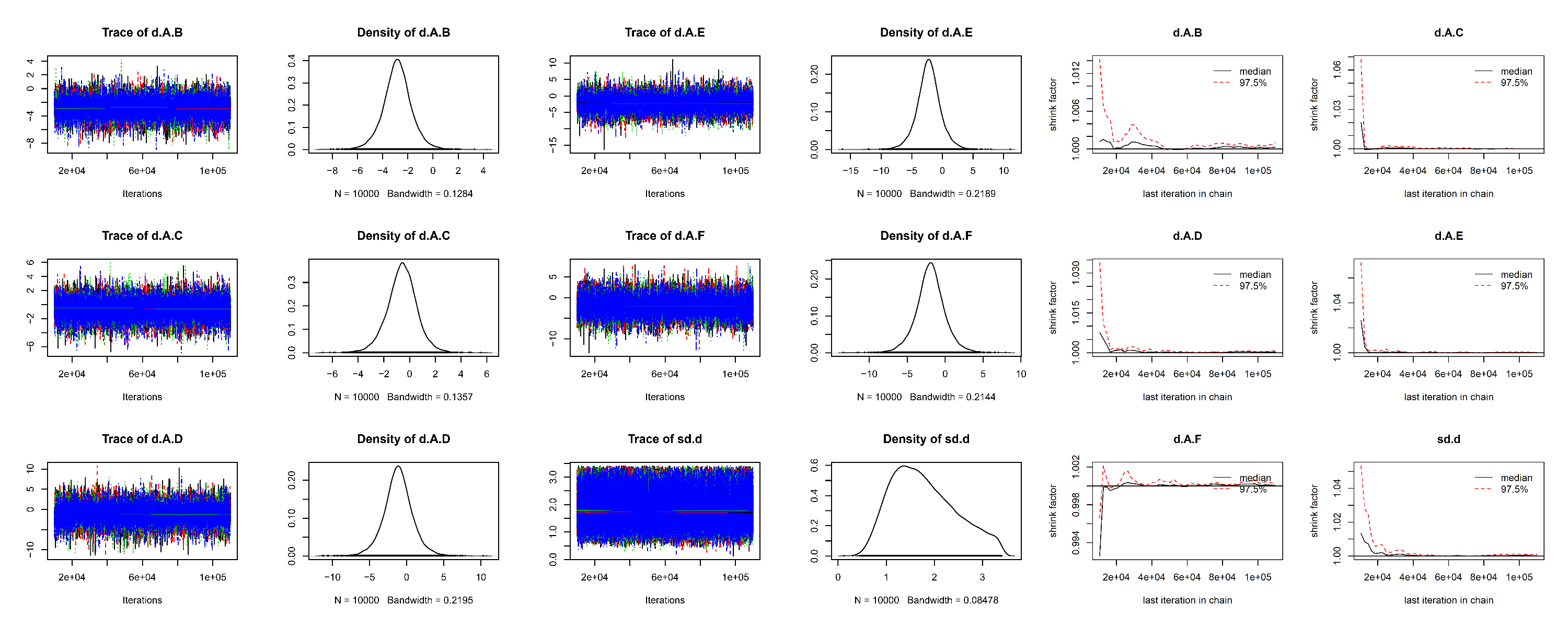


**Treatments of the various groups: A represents the group treated with mecobalamine; B represents mecobalamine combined with acupuncture; C represents mecobalamine combined with TCM foot bat; D represents mecobalamine combined with acupoint application; E represents mecobalamine combined with acupoint injection; F represents mecobalamine combined with TCM fumigation.**

**Fig S2 The trace map, density map and convergent diagnostic diagram of median nerve sensory conduction velocity**


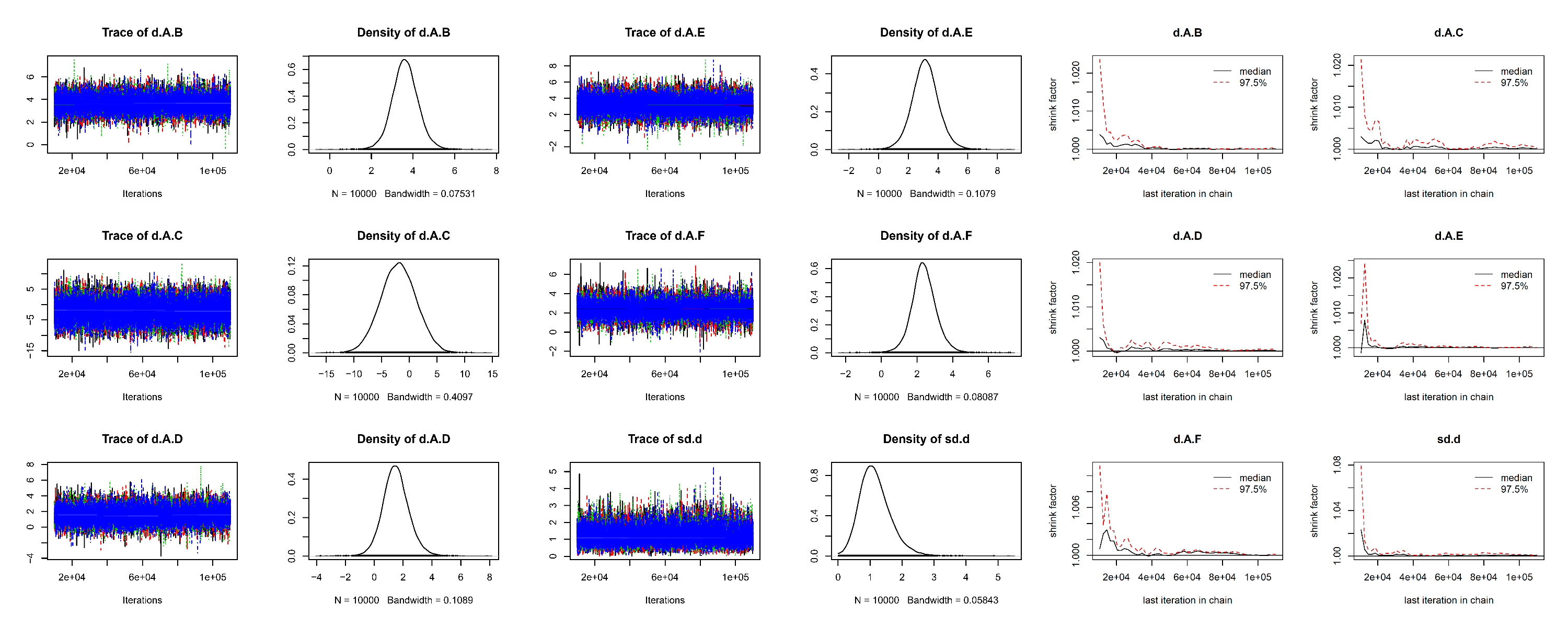


**Treatments of the various groups: A represents the group treated with mecobalamine; B represents mecobalamine combined with acupuncture; C represents mecobalamine combined with TCM foot bat; D represents mecobalamine combined with acupoint application; E represents mecobalamine combined with acupoint injection; F represents mecobalamine combined with TCM fumigation.**

**Fig S3 The trace map, density map and convergent diagnostic diagram of median nerve motor conduction velocity**


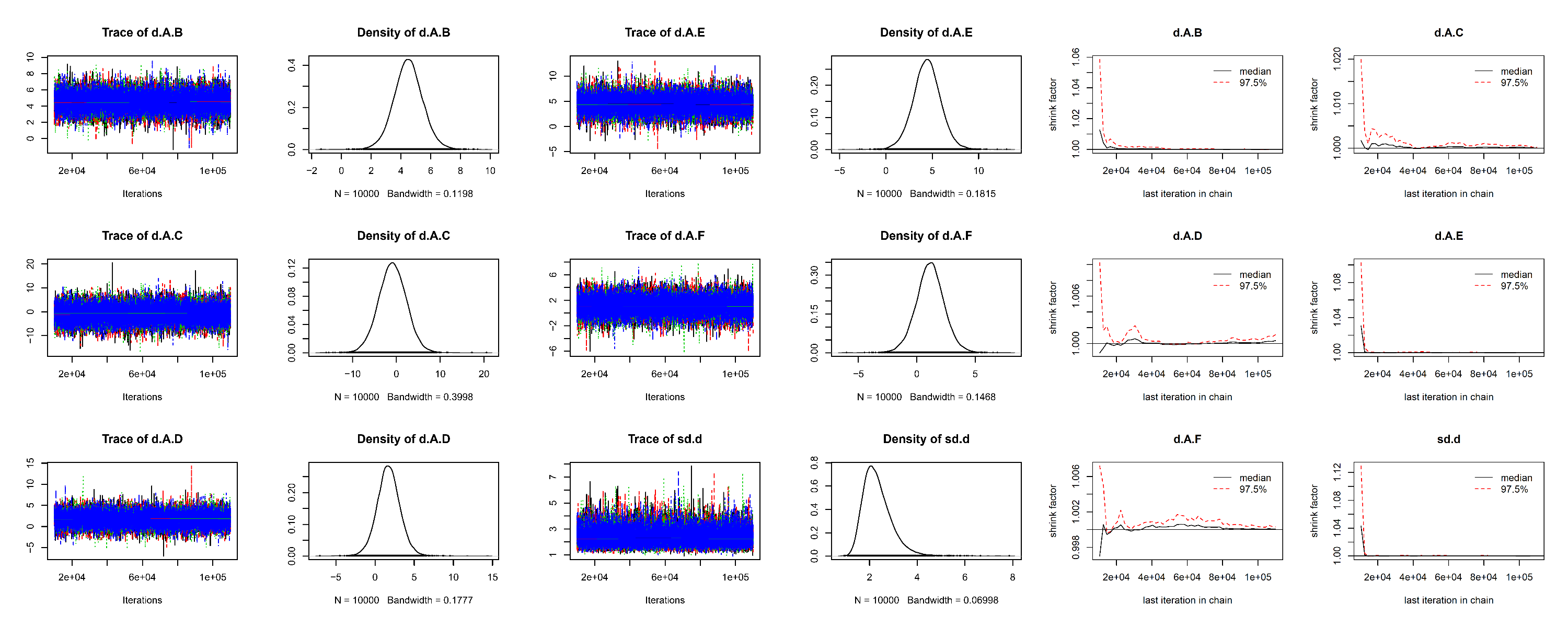


**Treatments of the various groups: A represents the group treated with mecobalamine; B represents mecobalamine combined with acupuncture; C represents mecobalamine combined with TCM foot bat; D represents mecobalamine combined with acupoint application; E represents mecobalamine combined with acupoint injection; F represents mecobalamine combined with TCM fumigation.**

Reference:

[1]Wang Dong. Clinical study of Huoluokangbi Decoction combined with mecobalamine in the treatment of diabetic peripheral neuropathy[D]. Inner Mongolia Medical University

[2]Zhang Yi,Zhang Min. Clinical observation on treating diabetic peripheral neuropathy with activating blood, dispelling wind and dehumidifying Chinese medicine foot bath combined with oral mecobalamin tablets[J]. Chinese journal of information on traditional chinese medicine,2014,21(12):22-24.

[3]Du MinZhen,Dong Jian,Huang Zaiqing,Zhang Dawei. Effects of warm acupuncture on nerve conduction and blood glucose metabolism in patients with diabetic peripheral neuropathy[J]. World Chinese Medicine,2019,14(11):3009-3012.

[4]Chen Guilian,Pan Hua. Effects of warm acupuncture on hemodynamics and nerve conduction velocity in diabetic peripheral neuropathy[J].Chinese Journal of Convalescent Medicine,2021,30(02):165-167.

[5]Yang Qijun. 84 cases of diabetic peripheral neuropathy treated by acupoint injection[J]. clinical research of TCM,2014,6(12):70-72.

[6]Xu Xiumei,Xiong Wenhua. Treatment of 30 cases of diabetic peripheral neuropathy with Qi deficiency and collateral obstruction by Yiqi Juanbi ointment combined with mecobalamine[J]. Journal of Jiangxi University of Chinese Medicine,2017,29(01):48-50.

[7]Han Liyun,Wu Qingmin,Yuan Yuxin,Yang Yujie. Clinical observation of acupuncture combined with mecobalamin tablets in the treatment of diabetic peripheral neuropathy[J]. Hebei Journal of Traditional Chinese Medicine,2016,38(02):246-248+256.

[8]Wang Junbao. Clinical observation of acupuncture combined with mecobalamin in the treatment of diabetic peripheral neuropathy[J]. Journal of Appropriate Clinical Medication,2018,11(05):28-29.

[9]Wang Zheng,Li Yanfang,Ma Ming. Effect of acupuncture combined with mecobalamin on diabetic peripheral neuropathy and its effect on serum inflammatory cytokines and plasma homocysteine[J]. Modern Journal of Integrated Traditional Chinese and Western Medicine,2018,27(14):1550-1553.

[10]WuFanghua,Zhu Qiyu. Clinical study of combined acupuncture and medicine in the treatment of diabetic peripheral neuropathy[J].Journal of Clinical Acupuncture and Moxibustion,2017,33(01):4-7.

[11]Chen Fang,Zhao Lu,Guo Xinxin. Clinical observation on 44 cases of diabetic peripheral neuropathy treated by integrated Traditional Chinese and Western medicine[J].Chinese Journal of Ethnomedicine and Ethnopharmacy,2020,29(12):108-112.

[12]Zhou Yi,Qiu Yingming. 48 cases of diabetic peripheral neuropathy treated by Antang fumigation and washing prescription[J]. Fujian Journal of Traditional Chinese Medicine,2015,46(04):30-32.

[13]Liu Liu,Jiang Chao,Zhao Zihao. Therapeutic effect of Salvia miltiorrhiza ligustrazine injection combined with Mecobalamin on patients with diabetes peripheral neuropathy and its effect on oxidative stress response[J]. Chinese Traditional and Herbal Drugs,2019,50(11):2670-2674.

[14]Jin Zhu,Wang Qiuyue,Huang Li,Ding Lu,Yao Qunying,Fan Yibin,Wang Siyao,Fei Zhujue. Clinical effect of hand-foot-warm acupuncture on diabetic peripheral neuropathy and its influence on nerve conduction velocity[J]. Hebei Journal of Traditional Chinese Medicine,2020,42(09):1374-1378.

[15]Zhou Yi,Qiu Yingming,Ye Xiangrong. Clinical observation on treatment of diabetes peripheral neuropathy with Tangbi plaster[J]. Traditional Chinese Medicine Journal,2018,17(06):47-50.

[16]Wang Kuigang,Zhang Ying. Study on the therapeutic effect of integrated traditional Chinese and Western Medicine on diabetes peripheral neuropathy[J]. Cardiovascular Disease Journal of Integrated Traditional Chinese and Western Medicine(Electronic) ,2015,3(28):42-43.

[17]Wang Zhong,Yu Hai,Zhang Chuan,Zong Xueping,Liang Qingyue,Li Chaomin. A randomized controlled study of traditional Chinese medicine fumigation combined with mecobalamin capsules on peripheral neuropathy in patients with diabetes [J]. Jilin Medical Journal

[18]Gao Mingsong,Peng Cong,Tan Yi. Treatment of diabetic peripheral neuropathy with Chinese herbal fumigation and mecobalamine: a summary of 42 cases[J]. Guiding Journal of Traditional Chinese Medicine and Pharmacology,2011,17(5):52-53.

[19]Zeng Douyun,Yan Guofu,Chen Huanling,Wu Quhui. 30 cases of diabetes peripheral neuropathy treated by fumigating and washing with traditional Chinese Medicine[J]. Chinese Medicine Modern Distance Education of China,2017,15(20):79-81.

[20]Liu Fan,Han Lin,Yang Meng. Clinical observation of 60 cases of diabetic peripheral neuropathy treated by Chinese herbal foot bath[J]. Journal of Jiangxi College of Traditional Chinese Medicine,2017,29(01):45-47.

[21]Han Qing,Wang Feng,Gao Pengfei,Zhang Yingjie,Xu Jia. Clinical observation on acupuncture combined with Mecobalamin acupoint injection in the treatment of senile diabetes peripheral neuropathy[J]. Geriatrics & Health Care,2018,24(04):442-445.

[22]Wu Yan. Clinical study on diabetes peripheral neuropathy treated with Quyu Tongluo XunXi Recipe[D]. Hubei University of Chinese Medicine,2011.
